# A Hand-Book for Nurses

**Published:** 1900-11

**Authors:** 


A HAND-BOOK FOR NURSES.—By J. K. Watson, M. D., Edn., Late House Surgeon, Essex and Colchester Hospital, etc., London. American edition, under the supervision of A. A. Stevens, A. M., M. D., Professor of Pathology in the Woman's Medical College of Pennsylvania, etc., Philadelphia. 413 pages. Price, $1.50. Philadelphia: W. B. Saunders, 925 Walnut Street. 1900.

This book is elaborate enough and still does not over-do the subject. It is admirably suited to training schools. It embraces in the one volume the whole subject. Its arrangement is convenient, and its language plain and free from unnecessary technicalities.

